# Comment on Utsumi et al. Differences in Pathophysiology and Treatment Efficacy Based on Heterogeneous Out-of-Hospital Cardiac Arrest. *Medicina* 2024, *60*, 510

**DOI:** 10.3390/medicina60081213

**Published:** 2024-07-26

**Authors:** Raghuraman M. Sethuraman, Buddhan Rajarathinam, Pranjali Kurhekar

**Affiliations:** 1 Department of Anesthesiology, Sree Balaji Medical College & Hospital, BIHER, Chennai 600044, India; 2 Department of Critical Care, Kauvery Hospital, Vadapalani, Chennai 600026, India

**Keywords:** neuron-specific enolase, shockable rhythm, non-shockable rhythm, out-of-hospital cardiac arrest

## Abstract

We read with great interest the review article on pathophysiology and treatment based on different out-of-hospital cardiac arrest (OHCA) patients. We wish to present our comments on the threshold values for neuron-specific enolase (NSE) based on the initial rhythm and the misquoting of a few references in that article.

We read with great interest the review article on pathophysiology and treatment based on different out-of-hospital cardiac arrest (OHCA) patients. We compliment Utsumi et al. [1] for this comprehensive review and wish to provide our comments.

While discussing the role of serum neuron-specific enolase (NSE) for the neurological outcomes in shockable and non-shockable OHCA patients, Utsumi et al. state that the cut-off value of NSE with a false positive rate ratio of <1% is also different for the non-shockable, shockable groups “(69.3 [sensitivity 42.1%] vs. 102.7 [sensitivity 76%] ng/mL, respectively)” [1]. This was stated based on a recently published article by Kim et al. [2]. However, in our view, Kim et al. [2] misinterpreted their results. As the median value of the shockable group was significantly lower than the non-shockable group (25.9 vs. 104.6) [2], it is not possible to have a higher cut-off for the shockable group. Hence, the correct NSE cut-off value should be 102.7 ng/mL for the non-shockable rhythm and 69.3 ng/mL for the shockable rhythm, and not vice versa. Furthermore, the sensitive values were also interchanged. It should have been 76% for the non-shockable and 42.1% for the shockable group. In addition, there were also some inconsistencies while reporting the results (sensitivity, false-positive ratio) in that study. Importantly, this is not a simple typographical error made by Kim et al. [2] as their discussion and conclusions are also based on this misinterpretation and thus could potentially mislead the researchers as is the case with Utsumi et al. [1].

Additionally, Utsumi et al. [1] misquoted the referenced article by Kim et al. [2] (cited as reference # 24 in the review article of Utsumi et al. [1]) while discussing the role of adrenaline for treatment during resuscitation. Kim et al. analyzed the impact of initial rhythm on the prognostic values of serum NSE [2], as mentioned earlier, and did not study the effects of adrenaline.
